# A Novel Role for *Helicobacter pylori* Gamma-Glutamyltranspeptidase in Regulating Autophagy and Bacterial Internalization in Human Gastric Cells

**DOI:** 10.3390/cancers11060801

**Published:** 2019-06-10

**Authors:** Jimena Bravo, Paula Díaz, Alejandro H. Corvalán, Andrew F.G. Quest

**Affiliations:** 1 Laboratory of Cellular Communication, Center for the Study of Exercise, Metabolism and Cancer (CEMC), Program in Cell and Molecular Biology, Institute of Biomedical Sciences (ICBM), Faculty of Medicine, Universidad de Chile, Santiago 8380453, Chile; jimenabravoortega@gmail.com (J.B.); pdiaztm@gmail.com (P.D.); 2 Advanced Center for Chronic Diseases (ACCDiS), Santiago 8380492, Chile; acorvalan@uc.cl; 3 Laboratory of Oncology, Department of Hematology and Oncology, Pontificia Universidad Católica de Chile, Santiago 8330034, Chile

**Keywords:** *Helicobacter pylori*, gamma-glutamyltranspeptidase, gastric cancer, autophagy, internalization

## Abstract

The risk of developing gastric cancer is strongly linked to *Helicobacter pylori* (*H. pylori*) infection. Alternatively, autophagy is a conserved response that is important in cellular homeostasis and provides protection against bacterial infections. Although *H. pylori* is typically considered an extracellular bacterium, several reports indicate that it internalizes, possibly to avoid exposure to antibiotics. Mechanisms by which *H. pylori* manipulates host cell autophagic processes remain unclear and, importantly, none of the available studies consider a role for the secreted *H. pylori* virulence factor gamma-glutamyltranspeptidase (HpGGT) in this context. Here, we identify HpGGT as a novel autophagy inhibitor in gastric cells. Our experiments revealed that deletion of HpGGT increased autophagic flux following *H. pylori* infection of AGS and GES-1 gastric cells. In AGS cells, HpGGT disrupted the late stages of autophagy by preventing degradation in lysosomes without affecting lysosomal acidification. Specifically, HpGGT impaired autophagic flux by disrupting lysosomal membrane integrity, which leads to a decrease in lysosomal cathepsin B activity. Moreover, HpGGT was necessary for efficient internalization of the bacteria into gastric cells. This important role of HpGGT in internalization together with the ability to inhibit autophagy posits HpGGT as a key virulence factor in the development of gastric cancer.

## 1. Introduction

Gastric cancer (GC) is the third leading cause of death due to cancer worldwide [1]. The major risk factor associated with the development of gastric cancer is colonization and chronic infection by the bacterial pathogen *Helicobacter pylori* (*H. pylori*), a Gram-negative bacterium that colonizes the gastric epithelium of ~50% of the world population. The pathogenicity of *H. pylori* is attributed to multiple virulence factors, including urease, catalase, peptidoglycan, neutrophil-activating protein (NapA), cytotoxin-associated-gene A (CagA), the cag pathogenicity island (cag PAI), vacuolating toxin (VacA), and the outer membrane proteins like the sialic acid-binding adhesin (SabA), blood group antigen binding adhesin (BabA), adherence-associated lipoprotein (AlpA) and outer membrane inflammatory protein (OipA). Among these, CagA and VacA are the best characterized virulence factors and both increase the risk for developing gastric cancer [2,3]; however, more recently, other important pathogenic factors that contribute to virulence of the bacterium have been described, one such factor being *H. pylori* gamma-glutamyltranspeptidase (HpGGT) [4]. GGT is an enzyme that catalyzes the transpeptidation and hydrolysis of the γ-glutamyl moiety of glutathione and glutathione-conjugated compounds, to amino acids [5]. HpGGT is constitutively expressed and is commonly found in all *H. pylori* strains [6], suggesting it plays an important role in the physiology of the bacterium. Among the multiple effects in gastric cells, GGT has been found to induce apoptosis by a mitochondria-dependent pathway [7] and also to reduce cell viability, as well as cause cell death by decreasing survivin levels [8], inducing cell cycle arrest [9], the generation of reactive oxygen spicies (ROS), in particular H_2_O_2_, leading to glutathione depletion and DNA damage [10].

Autophagy is a catabolic process important in maintaining cellular homeostasis that also provides protection against bacterial infections [11]. Several intracellular pathogens, such as *Mycobacterium tuberculosis*, *Salmonella typhimurium*, *Listeria monocytogenes*, among others, have been shown to regulate autophagy using different strategies to benefit from the process [12]. *H. pylori* reportedly can induce or prevent autophagy via the virulence factor VacA in gastric epithelial cells and the outcome appears to depend on whether cells are infected for short or extended periods, respectively [13,14]. Although, *H. pylori* is typically considered an extracellular bacterium, several studies have reported that it may be internalized, possibly as a strategy to avoid exposure to antibiotics [15,16,17]. Indeed, intracellular survival of *H. pylori* can be increased by down- or upregulation of microRNAs [18,19]. Interestingly, a recent study has shown that *H. pylori* increases survival by preventing its degradation in the lysosomes [20]. Although most of the studies in the literature point towards VacA as the only virulence factor involved in *H. pylori*-mediated regulation of autophagy, information concerning how precisely VacA regulates this process remains controversial, suggesting that additional *H. pylori* virulence factors might be implicated. Here, we provide evidence suggesting a novel role for HpGGT in regulating autophagy.

## 2. Results

### 2.1. Helicobacter Pylori Gamma-Glutamyltranspeptidase Inhibits Autophagy in Human Gastric Cancer Cells

**Figure 1:**
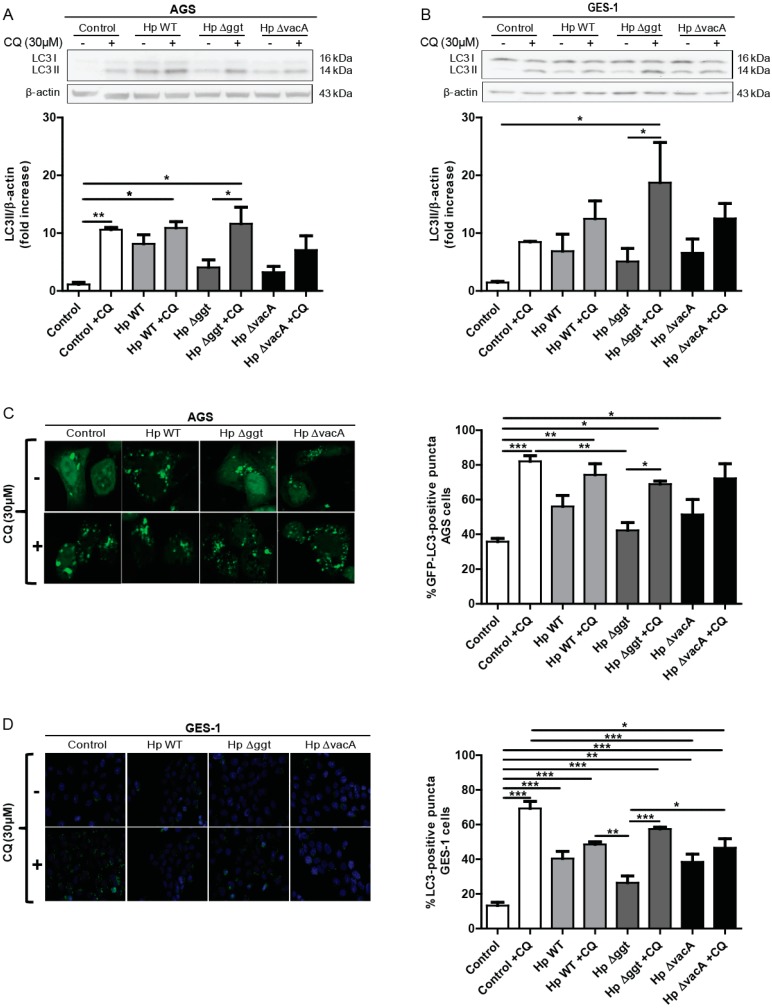
The isogenic mutant Δggt, lacking gamma-glutamyltranspeptidase (GGT), increases autophagic flux after infection of AGS and GES-1 cells when compared with the parental and the isogenic mutant HpΔvacA (lacking vacuolating toxin) strains. (**A**) AGS and (**B**) GES-1 cells were infected with wild type (HpWT) or the isogenic mutants Hp∆ggt and Hp∆vacA for 6 h in the presence or absence of chloroquine (CQ) (30 μM). Protein levels of the microtubule-associated protein 1A/1B light chain 3 (LC3) conjugated to phosphatidylethanolamine (LC3-II) and β-actin were evaluated by western blotting. To quantify the accumulation of autophagosomal structures in the presence or absence of CQ, (**C**) AGS cells were transiently transfected with the green fluorescent protein (GFP)-LC3-encoding plasmid and (**D**) LC3 endogenous expression was assessed by immunofluorescence in GES-1 cells. Infected cells were imaged for GFP-LC3 and LC3 puncta by confocal microscopy (AGS cells) and fluorescence microscopy (GES-1 cells). Autophagic structures were considered to be positive when the number of GFP-LC3 or LC3 puncta per cell was at least 5 (≥100 cells per experiment). These data represent the mean ± the standard error of the mean (SEM) of three independent experiments. * *p* < 0.05, ** *p* < 0.01, *** *p* < 0.001.

To evaluate whether HpGGT modulates autophagy, two gastric cell lines—AGS and GES-1—were infected for 6 h at a multiplicity of infection (MOI) of 100 with the wild type *H. pylori* strain 26695 or the respective isogenic Hp∆ggt and Hp∆vacA mutant strains. Among other proteins, the lipidated levels of the microtubule-associated protein 1A/1B-light chain 3 (LC3) conjugated to phosphatidylethanolamine (LC3-II) are widely used to monitor autophagic activity. However, due to the dynamic nature of this process, increased levels of LC3-II (Western blot analysis) or an accumulation of green fluorescent protein (GFP)-LC3 puncta (confocal analysis of cells transfected with a plasmid encoding GFP-LC3) are indicative of either the induction of autophagy or a block in autophagosome fusion or decreased lysosomal degradation [21]. Given this ambiguity in the interpretation of results, we evaluated the autophagic flux by determining autophagosome accumulation after 6 h in the presence or absence of the lysosomal degradation inhibitor chloroquine (CQ). In both cell lines, we observed for the isogenic mutant Hp∆ggt (Figure 1A,B) that LC3-II levels were significantly higher in the presence of CQ than without CQ, indicating increased autophagic flux. However, for neither the parental (HpWT) nor the Hp∆vacA mutant strain did autophagic flux increase significantly. 

Then, the accumulation of autophagosomal structures after *H. pylori* infection (MOI 100, 6 h) in the presence or absence of CQ was assessed by transient transfection of AGS cells with the GFP-LC3-encoding plasmid, while endogenous LC3 expression was determined in GES-1 cells. The number of GFP-LC3 (AGS) and LC3 (GES-1) positive cells (>5 dots per cell) was determined by confocal microscopy. As expected, a significant increase in the number of GFP-LC3 positive cells was detected in the presence of CQ in those cells infected with the isogenic mutant Hp∆ggt but not when infected with either HpWT or the Hp∆vacA strain (Figure 1C,D). These results indicate that in the presence of the virulence factor GGT (in HpWT and Hp∆vacA bacteria) autophagic flux is decreased and therefore that autophagy is being inhibited in AGS and GES-1 gastric cells.

### 2.2. HpGGT Activity in the Supernatants of H. pylori Cultures Was Sufficient to Inhibit Autophagy of Gastric Cells

**Figure 2:**
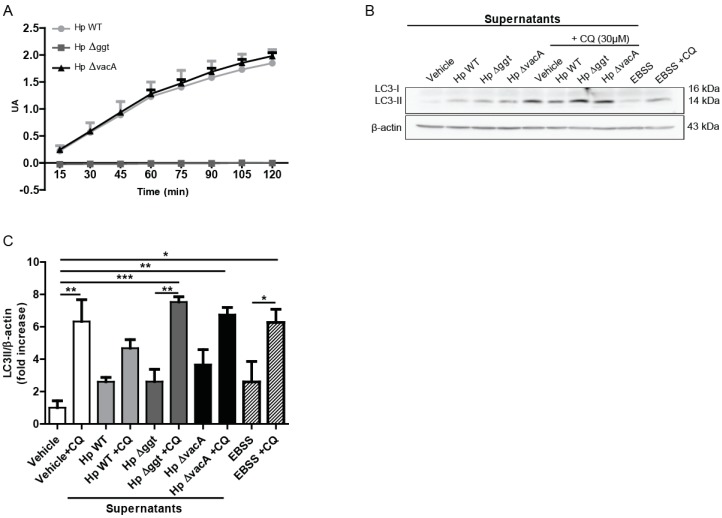
Hp∆ggt culture supernatant increases autophagic flux of AGS cells in comparison with the parental strain. (**A**) Activity of gamma-glutamyltranspeptidase in cell lysates of wild type (HpWT) and the isogenic Hp∆ggt and Hp∆vacA mutants (lacking gamma-glutamyltranspeptidase and vacuolating toxin, respectively). (**B**) AGS cells were incubated for 6 h with the concentrated supernatants from wild type, Hp∆ggt and Hp∆vacA *H. pylori* strains (1:25) in the presence or absence of chloroquine (30 μM). As a control, concentrated non-conditioned medium (vehicle) was added at a similar dilution. In addition, Earle’s Balanced Salt Solution medium (amino acid and serum free medium) was used as a positive control to induce autophagy (4 h). Protein levels of the microtubule-associated protein 1A/1B light chain 3 (LC3) conjugated to phosphatidylethanolamine (LC3-II) and β-actin were evaluated by Western blotting. In panel **C**) the quantification of relative levels of LC3-II for AGS cells is shown. These data represent the mean ± the standard error of the mean (SEM) of three independent experiments. * *p* < 0.05, ** *p* < 0.01, *** *p* < 0.001.

Because HpGGT is a secreted virulence factor, we also evaluated whether GGT activity present in *H. pylori* culture supernatants might promote changes in autophagy of AGS cells without the need for direct bacterial contact. As we observed with the bacteria, Hp∆ggt culture supernatants (without GGT activity, Figure 2A) increased autophagic flux of AGS cells in comparison with the parental strain (Figure 2B,C). In addition, some differences in the levels of LC3-II following treatment with CQ were observed for *H. pylori* supernatants in comparison with those obtained with the bacteria, suggesting that although the GGT present in *H. pylori* culture supernatants is able to reduce autophagic flux in AGS cells, the presence of the bacteria can additionally modulate this response. 

### 2.3. HpGGT Did Not Affect the Early Steps of Autophagy

In order to elucidate how HpGGT inhibits autophagy, we first evaluated whether this virulence factor regulated the autophagic pathway at the level of induction and formation of autophagosomes by inhibiting mTOR (mammalian target of rapamycin) or AMPK (Thr 172, protein kinase, AMP activated) activity. The two protein kinases—mTOR and AMPK—are known to regulate the early steps of autophagy by either inhibition or activation of the process, respectively (canonical pathway).

We did not observe any alterations in the phosphorylation of either the downstream targets of mTOR (Figure S1A) or AMPK (Figure S1B) after infection of AGS cells with *H. pylori* wild type or the isogenic Hp∆ggt mutant strain. These results allow us to conclude that HpGGT inhibits autophagy without modulating the canonical AMPK or mTOR pathways in AGS cells.

### 2.4. HpGGT Contributes to Autophagy Inhibition without Modifying Lysosome Acidification

**Figure 3:**
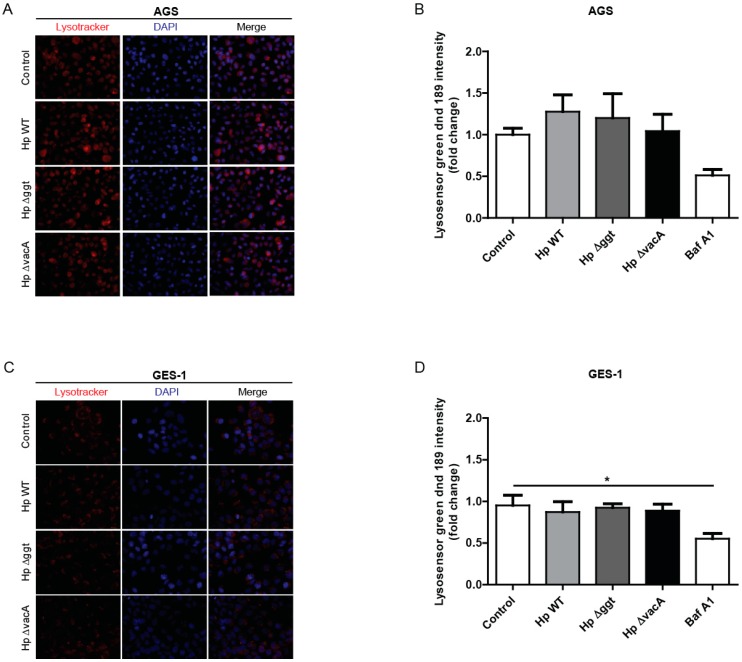
*H. pylori* infection did not affect lysosomal acidification in gastric cells. AGS and GES-1 cells were infected with *H. pylori* wild type (HpWT) or the isogenic Hp∆ggt and Hp∆vacA (lacking gamma-glutamyltranspeptidase and vacuolating toxin, respectively) mutant strains at a multiplicity of infection (MOI) of 100 for 6 h. (**A**) To visualize acidified compartments, (**A**) AGS and (**C**) GES-1 cells were treated with the acidotropic dye Lysotracker Red DND-99 (75 nM) for 2 h. Representative fluorescent images from three independent infection experiments are shown. To measure the lysosomal pH, (**B**) AGS and (**D**) GES-1 cells were treated with Lysosensor green DND-189 (1 µM) for 30 min. Fluorescence intensity was evaluated by flow cytometry. Bafilomycin A1 (100 nM) was used as positive control to inhibit acidification. Data represent the mean ± the standard error of the mean (SEM) of at least three independent experiments. * *p* < 0.05.

LC3-II levels are closely correlated with the number of autophagosomes, which serves as a good indicator of autophagosome formation [22]. We have shown that the levels of LC3-II in the presence of CQ were very similar following infection with the parental strain and the Hp∆vacA mutant, but not with the Hp∆ggt mutant, for which we observed higher levels (Figure 1A,B,D). Therefore, we examined whether GGT disrupted the late stages of autophagy by preventing degradation in lysosomes. To this end, we evaluated the activity of the lysosomal compartment by using the cell-permeable acidotropic probe LysoTracker® Red, which selectively labels vesicles with low internal pH. As shown in Figure 3A,C, AGS and GES-1 cells infected with either HpWT or the isogenic Hp∆ggt and Hp∆vacA mutants induced the accumulation of acidic vacuoles to a similar extent. Furthermore, because acidic pH is required for lysosomal activity, we used the Lysosensor green DND-189 dye to assess whether HpGGT affected lysosomal pH. As shown in Figure 3B,D, there were no significant differences in lysosomal acidity between infected and control cells. Thus, these results indicate that impaired autophagic flux by HpGGT was not due to inhibition of lysosomal acidification.

### 2.5. HpGGT Inhibits Autophagy of Gastric Cells by Decreasing Cathepsin B Activity in the Lysosome

**Figure 4:**
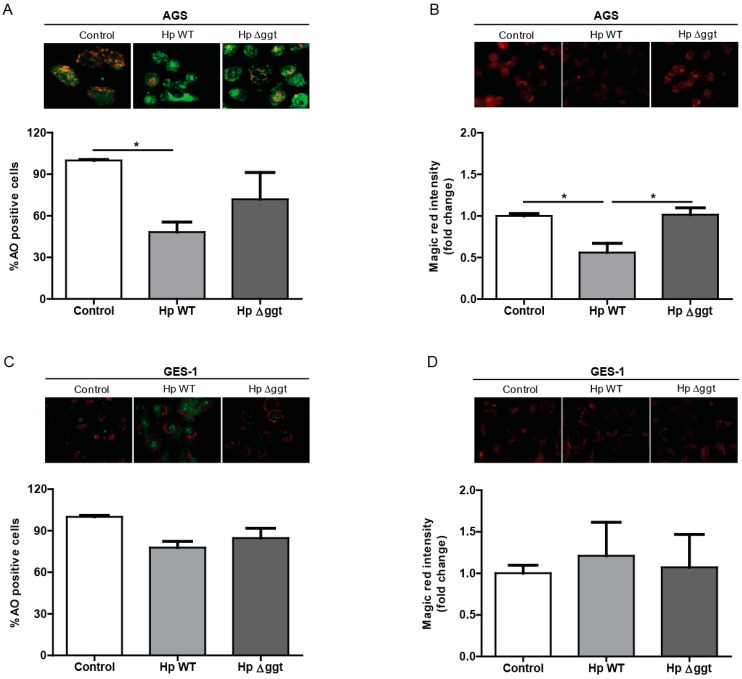
HpGGT inhibits autophagy of AGS cells by decreasing cathepsin B activity in the lysosome. (**A**) AGS and (**C**) GES-1 cells were infected with *H. pylori* wild type (HpWT) and the isogenic Hp∆ggt mutant (lacking gamma-glutamyltranspeptidase) at a multiplicity of infection (MOI) of 100 for 6 h. Lysosomal membrane integrity was determined by measuring Acridine Orange (AO) staining in a fluorescence microscope. Cells were treated with AO 5 µg/mL for 15 min. The quantification of AO positive cells (>5 red puncta per cell) is shown. To measure cathepsin B activity, live (**B**) AGS and (**D**) GES-1 cells were stained with the Cathepsin B substrate Magic Red^TM^ for 15 min and staining intensity was evaluated by fluorescence microscopy. Data represent the mean ± the standard error of the mean (SEM) of at least three independent experiments. Representative images are shown. * *p* < 0.05.

Next, we determined whether the function of the lysosomal compartment was affected by HpGGT. Because accumulation of undigested autophagosomes may result in lysosomal permeabilization (LMP) we evaluated lysosome membrane integrity by Acridine Orange (AO) staining. As shown for AGS cells in Figure 4A, after 6 h of infection with HpWT and the isogenic Hp∆ggt mutant, the number of AO positive cells (>5 red puncta per cell) was significantly lower in comparison with control cells. This suggests that *H. pylori* infection induced significant lysosomal membrane permeabilization in AGS. However, this appeared not to be the case for GES-1 cells (Figure 4C).

Therefore, taking into account that after *H. pylori* infection we observed LMP, we evaluated whether the activity of cathepsin B, a prevalent lysosomal protease decreased. Cathepsin B activity was measured by fluorescence microscopy after Magic Red^TM^ staining. As indicated for AGS cells in Figure 4B, the fluorescence signal of Magic Red following infection with HpWT bacteria was significantly lower than that for the Hp∆ggt strain, indicating that the proteolitic activity of cathepsin B was reduced after *H. pylori* infection and that HpGGT contributed to this effect. However, after *H. pylori* infection of GES-1 cells (Figure 4D), we did not observe significant changes in cathepsin B activity in comparison with control cells.

### 2.6. Loss of HpGGT Decreases H. pylori Internalization in Gastric Cells

**Figure 5:**
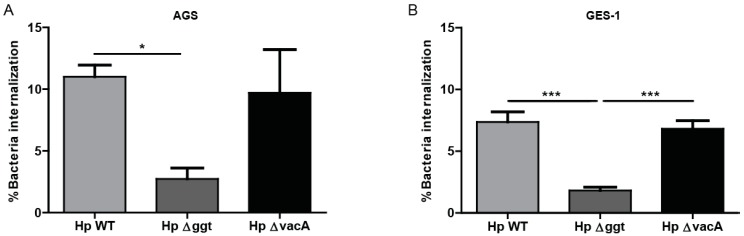
Loss of HpGGT decreases *H. pylori* internalization in gastric cells. AGS and GES-1 cells were infected with *H. pylori* wild type (HpWT) and the isogenic mutants Hp∆ggt and Hp∆vacA (lacking gamma-glutamyltranspeptidase and vacuolating toxin, respectively) at a multiplicity of infection (MOI) of 100 for 6 h. *H. pylori* internalization was evaluated using the colony unit forming assay. After infection, cells were treated with gentamycin (200 µg/mL) for 1 h in order to eradicate extracellular bacteria. The percentage of intracellular viable *H. pylori* is shown. These data represent the mean ± the standard error of the mean (SEM) of three independent experiments. * *p* < 0.05, *** *p* < 0.001.

Finally, we evaluated the possibility that HpGGT inhibits autophagy as a strategy to survive after cell infection. To this end we assayed the internalization of HpWT bacteria and the isogenic Hp∆ggt and Hp∆vacA mutants in AGS and GES-1 cells. As shown in Figure 5, the mutant Hp∆ggt (lacking GGT) displayed a significantly reduced capacity to internalize into AGS and GES-1 cells than HpWT bacteria.

## 3. Discussion

The association between *H. pylori* and autophagy in the context of gastric cancer has been extensively reviewed [23,24,25]; however, several questions remain concerning the precise mechanisms by which the bacterium modulates this catabolic process. Although the existing literature related to autophagy and *H. pylori* infection suggests that the virulence factor VacA is the main modulator of the autophagic process in host cells after *H. pylori* infection, the controversial data point towards a role for additional virulence factors. In the present study, we identified the secreted virulence factor GGT as a novel autophagy inhibitor. In our in vitro model, the *H. pylori* strain lacking GGT (Hp∆ggt) failed to inhibit autophagy to the same extent as the wild type strain after 6 h of infection. Interestingly, consistent with the fact that GGT is a secreted virulence factor, we found that autophagic flux also decreased after the treatment of AGS cells with concentrated supernatants obtained from HpWT bacteria, but not the mutant strain lacking GGT. Furthermore, considering that Terebiznik and collaborators [13] reported on the effects of VacA in *H. pylori*-mediated autophagy after 6 h of AGS cell infection, we also evaluated the mutant strain Hp∆vacA, lacking VacA, under the same conditions. In agreement with these authors, lack of VacA prevented autophagy induction. Nevertheless, in our experimental conditions, we did not observe the induction of autophagy with HpWT bacteria, expressing VacA and GGT. In the present study, the LC3-II levels in the presence of CQ were very similar in AGS and GES-1 cells after infection with the wild type strain and the isogenic Hp∆vacA mutant but not after infection with the Hp∆ggt mutant. Based on the aforementioned notion that LC3-II levels are a good indicator of autophagosome formation [22], we suggest the existence of a functional connection between the virulence factors VacA and GGT. Thus, in early stages of infection with *H. pylori*, VacA may induce autophagy (higher LC3-II levels in presence of CQ in *H. pylori* strains without GGT but with VacA such as Hp∆ggt), but at late stages, such as lysosome degradation, GGT appears to inhibit autophagy. Although the association between VacA and GGT is not as well established as that between VacA and CagA [26], Ling and collaborators [27] reported in gastric epithelial cells that GGT enhanced the formation of vacuoles, one of the major VacA effects. These connections between the different virulence factors need to be considered when the effects of *H. pylori* induced autophagy are interpreted. Thus, additional experiments are required to clarify the details of this connection between GGT and VacA in modulating autophagy.

Since the GGT-mediated inhibition of autophagy in AGS cells could not be explained by the canonical pathways involving AMPK or mTOR [28] (Supplementary Figure S1), we evaluated the effect of GGT in the late stages of autophagy. The cargo selected for digestion is finally degraded in acidic organelles, called lysosomes, which contain hydrolases [29]. To evaluate lysosome activity, we labelled acidic vesicles with the fluorescent probe Lysotracker red dnd-99. Our results show that after 6 h of *H. pylori* infection, fluorescence intensity did not change when we compared control cells with infected cells. Because lysosome digestive function depends on appropriate acidification [30], we evaluated whether GGT affected the pH. In the present study, we did not find changes in lysosomal pH when we compared noninfected with infected cells. These findings are consistent with previous studies that also observed unaltered lysosomal pH after 2 h and 24 h of *H. pylori* infection [14,17]. Nevertheless, Zhang et al. [20] recently reported on an increase in lysosomal pH after *H. pylori* infection. These authors suggested that this discrepancy might be due to different experimental conditions. In fact, the biggest difference resides in the infection model. Zhang and collaborators [20] infected for only 3 h and eliminated extracellular bacteria with gentamicin. Then they maintained cells in culture for an additional 72 h. These discrepancies highlight the potential importance of the infection conditions when results are interpreted.

Considering that GGT did not affect lysosomal activity, we evaluated lysosomal membrane integrity using Acridine Orange. In our model, we found that *H. pylori* infection caused lysosomal membrane permeabilization in AGS cells and this effect was independent of GGT. In agreement with this result, Zhang et al. [20] also observed a decrease in red puncta fluorescence intensity after *H. pylori* infection. On the one hand, although LMP is closely related to cell death [31], we did not evaluate this parameter here because it is well known that longer periods of infection with *H. pylori* are necessary to cause cell death [32]. However, it is likely that following LMP, cells will not be able to remove damaged lysosomes and this can be expected to contribute to long-term cell death. On the other hand, ROS production is well established as a LMP precursor [33,34]. According to previous studies, *H. pylori* infection generates ROS in gastric epithelial cells and some of these studies point towards GGT as one of the virulence factors implicated [8,10,35]. Although we also observed LMP in the absence of GGT, we cannot rule out the possibility that LMP was caused by ROS production. To confirm this hypothesis additional studies are required. 

Because of LMP, we expected that some hydrolytic enzymes, such as cathepsins, might be released into the cytosol. Although we did not measure the presence of these enzymes in the cytoplasm, we checked whether GGT modified cathepsin B activity. Our results show that after infection with the *H. pylori* wild type strain cathepsin B activity decreased in AGS cells, and this effect was not observed upon infection with the Hp∆ggt strain (lacking GGT). In agreement with this result, previous studies also observed the presence of lysosomes with less hydrolytic activity [14,20]. In particular, Raju and collaborators [14] showed that VacA was responsible for these defective lysosomes. Considering that the final step of autophagy is degradation in the lysosomes, our results suggest that after *H. pylori* infection of AGS cells, GGT decreases cathepsin B activity preventing cargo digestion in the lysosomes and thereby blocks autophagy at these late stages. A caveat to this interpretation is that while we did observe that GGT contributed to inhibition of autophagy in GES-1 cells after *H. pylori* infection, this was not attributable to changes in cathepsin B activity. Thus, the precise mechanism by which autophagy is modulated by bacterial GGT in these cells remains to be determined.

Bearing in mind that autophagy represents a tumor suppressor mechanism in the early stages cancer by preventing the excessive generation of ROS, as well as ensuing mitochondrial and DNA damage, the inhibition of autophagy by HpGGT can be expected to cause genome instability in *H. pylori*-infected gastric epithelial cells, which in turn generates a favorable environment for carcinogenesis [36]. Beyond genetic changes, it has been extensively described that epigenetic alterations mediated indirectly or directly by *H. pylori* infection play a major role in gastric cancer development [37,38,39,40]. In this context, it was recently reported that *H. pylori*-mediated autophagy inhibition involves modifying the expression of the autophagy-related gene (*Atg*) variant MAP1LC3Av1. Indeed, the authors provide evidence for hypermethylation of MAP1LC3Av1 in the gastric mucosa tissues of *H. pylori*-infected individuals. Moreover, silencing of this *Atg* gene variant also reduced autophagy in gastric epithelial cells [41]. According to these results, Tanaka and collaborators [42], found that several genes implicated in regulating autophagy, such as *ATG16L1*, were downregulated in the gastric mucosa of *H. pylori* infected volunteers. All together, these observations highlight the importance of *H. pylori*-mediated inhibition of autophagy as a relevant mechanism in the progression of precancerous lesions to gastric cancer. However, further studies are required to determine whether and how the virulence factor HpGGT aids in promoting epigenetic changes during gastric cancer progression.

The notion that *H. pylori* might be a facultative intracellular bacteria is becoming of increasing interest [43]. Several studies have described that *H. pylori* can survive inside epithelial cells [15,16] and that this capacity of the bacteria might contribute to its pathogenesis and persistence [44]. In this context, we observed in the gentamicin protection assay that after 6 h of infection the percentage of internalized viable bacteria was reduced in the case of the mutant Hp∆ggt strain compared with the wild type strain. However, we did not observe changes in the percentage of bacteria internalization with the mutant Hp∆vacA strain. Consistent with these findings, Terebiznik et al. [17] also described that the virulence factor VacA was not required for entry into gastric epithelial cells. Interestingly, the number of adherent bacteria was very similar for all *H. pylori* strains (Supplementary Figure S2). Thus, lack of GGT did not affect the ability of *H. pylori* to adhere to the cell surface, but rather emerges as a key virulence factor involved in bacterial internalization. In agreement with this result, Wustner and collaborators [45] recently described that GGT is required mainly for initial colonization in mice. Thus, our results highlight the fact that GGT is not just important for bacterial metabolism, but also aids in *H. pylori* internalization into host cells and in doing so serves to avoid *H. pylori* exposure to antibiotics.

## 4. Material and Methods

### 4.1. Cell lines, Strains, and Culture Conditions

The gastric cancer cell lines AGS (ATCC^®^ CRL-1793™) were obtained from the American Tissue Culture Collection and the nontransformed cell line GES-1 (kindly provided by Dr. Armando Rojas, Universidad Católica del Maule, Chile). Cells were cultured in Roswell Park Memorial Institute medium (RPMI 1640 medium, Thermo Fisher Scientific, Waltham, MA, USA) supplemented with 10% Fetal Bovine Serum (Biological Industries, Cromwell, Connecticut, USA) and antibiotics (10,000 U/mL penicillin, 10 μg/mL streptomycin). Cells were incubated at 37° C in a humidified atmosphere with 5% CO_2_. The completely sequenced *H. pylori* strain 26695 (ATCC 700392) was employed here as the wild type strain. Isogenic mutant hp0887::aph (HpΔvacA), kindly provided by Dr Guillermo Pérez-Pérez (New York University), and the previously described isogenic mutant hp1118::cat (GGT; HpΔggt) [8] were also evaluated.

### 4.2. Infection of Gastric Cells with H. pylori 

For infection assays, gastric cells were infected with a multiplicity of infection (MOI) of 100 (bacteria: cell) in all experiments as described Valenzuela et al. [32]. The concentrated culture supernatants were incubated with gastric cells at a final dilution of 1:25 as previously described [8]. 

### 4.3. Concentrated Culture Supernatants 

The concentrated culture supernatants were prepared as described Valenzuela et al. [8]. GGT activity was measured according to the method described by Chevalier et al. [6]. Briefly, quantitative determination of GGT activity in concentrated bacterial culture supernatants was determined at 37 °C in 0.1 M Tris-HCl buffer (pH 8.0) using 1 µM L-glutamyl-p-nitroanilide as the donor substrate in the presence or absence of 20 µM glycylglycine as the acceptor. The absorbance of p-nitroaniline liberated from γ-glutamyl-p-NA was measured at 385 nm. Data was expressed as arbitrary absorbance units per mg protein.

### 4.4. Autophagosome Analysis 

For transient expression of the GFP-LC3-encoding plasmid, AGS cells were transfected in serum free medium with Lipofectamine® 2000 (Thermo Fisher Scientific, Waltham, Massachusetts, USA) according to the manufacturer’s instructions. However, in GES-1cells GFP-LC3 was difficult to detect, so we assessed endogenous LC3 endogenous expression instead. Afterwards, AGS and GES-1 cells were seeded onto 12 mm coverslips in 24-well plates. The cells were infected with wild type (HpWT) or the isogenic HpΔggt or HpΔvacA (lacking GGT and VacA, respectively) mutants for 6 h in the presence or absence of CQ (30 μM). Then, samples were prepared for indirect immunofluorescence analysis, as indicated below, evaluated by confocal (AGS cells) or fluorescence (GES-1 cells) microscopy. The cells positive for GFP-LC3 or LC3 puncta (>5 dots per cell) were determined using the ImageJ software (Rasband, W.S. (1997–2019) ImageJ. National Institutes of Health, Bethesda, Maryland, USA. http://imagej.nih.gov/ij).

### 4.5. Immunofluorescence (IF) and Confocal Microscopy

Cells were infected as described above and then were washed with ice-cold phosphate-buffered saline (PBS), fixed in paraformaldehyde (4%v/v) (Electron Microscopy Science, Hatfield, Pennsylvania, USA) or methanol at −20 °C (LC3 analysis), permeabilized with Triton 0.1% in PBS, and blocked in 3% bovine serum albumin (BSA)-PBS for 1 h at room temperature. Cells were stained with primary antibodies prepared in 3% BSA-PBS overnight at 4 °C. After that, secondary antibodies conjugated to AlexaFluor^®^ (Thermo Fisher Scientific, Waltham, Massachusetts, USA) were added for 1 h at room temperature. Nuclei were counterstained with DAPI (Sigma-Aldrich, D9542, St. Louis, Missouri, USA). Images were obtained with a C2 plus spectral confocal microscope (Nikon Eclipse TI (Tokyo, Japan) or using a fluorescence microscope (Spinning disk Olympus IX81) (Tokyo, Japan) as indicated.

### 4.6. LysoTracker Red Staining

AGS and GES-1 cells were infected and treated with 75 nM Lysotracker Red DND-99 (Thermo Fisher Scientific, Waltham, MA, USA) for 2 h. After washing, the samples were fixed with paraformaldehyde (4%v/v) and nuclei were counterstained with DAPI. The samples were viewed by fluorescence microscopy (Spinning disk Olympus IX81).

### 4.7. Flow Cytometry

Lysosomal pH was determined by flow cytometry using a FACSCanto flow cytometer (Becton Dickinson, New Jersey, USA) for noninfected and infected cells incubated with the Lysosensor green DND-189 (Thermo Fisher Scientific, Waltham, Massachusetts, USA) (1 µM) for 30 min.

### 4.8. Measurement of Lysosomal Membrane Stability 

Acridine Orange (AO, Bio-Rad, Hercules, California, USA) was used to evaluate lysosomal integrity. When lysosomes are intact AO exhibits red fluorescence (AO positive); however, when lysosomes are compromised and the lysosomal content diffuses into the cytosol, AO exhibits green fluorescence at low concentrations [46]. Gastric cells were seeded on coverslips in 6-well plates. Cells were infected for 6 h at MOI of 100. Then AGS and GES-1 cells were stained with 5 μg/mL AO at 37 °C for 15 min and then the cells were washed with PBS. Live cells were observed by fluorescence microscopy in Krebs-Ringer HEPES buffer. The number of AO positive cells (>5 red puncta per cell) was assessed using the Image J software.

### 4.9. Cathepsin B Activity (Magic Red)

Cathepsin B protease activity was determined using the commercial Cathepsin Magic Red^TM^ kit provided by Bio-Rad (Hercules, California, USA). After infection, cells were washed and incubated with Magic Red^TM^ MR-(RR)_2_ Reagent for 15 min according to the manufacturer’s protocol. Cathepsin B activity was assessed as staining intensity observed by fluorescence microscopy.

### 4.10. Adherence and Gentamicin Internalization Assays

AGS and GES-1 cells were cultured in 24-well tissue culture plates (1 × 10^5^ cells per well) and allowed to attach overnight. Prior to stimulation, the media was removed and replaced with fresh antibiotic-free media, and cells were incubated with *H. pylori* wild type or the respective isogenic HpΔggt and HpΔvacA mutants at a MOI of 100 for 6 h. After incubation, cells were washed at least five times with culture medium. For the internalization assay, cells were treated with gentamycin (200 µg/mL) for 1 h in order to eliminate extracellular *H. pylori*. Then, cells treated or not with gentamycin (total bacteria) were washed five times with PBS and lysed using saponin 0.1% for 15 min at 37 °C. Total and invaded bacteria were estimated by plating serial dilutions. The percent of intracellular bacteria was calculated by multiplying the number of viable intracellular bacteria by 100 and dividing by the total number of bacteria.

### 4.11. Western Blot Analysis

Cells were lysed in radioimmunoprecipitation assay (RIPA) buffer (50 mM Tris-HCl, pH 8.0; 150 mM NaCl, 1% NP-40 (IGEPAL), 0.5%, sodium deoxycholate and 0.1% sodium dodecyl sulphate) with both protease and phosphatase inhibitors. Total protein extracts (50 µg/lane) were separated in 15% SDS polyacrylamide gels and transferred to nitrocellulose membranes as described [8]. Membranes were then incubated with primary antibodies overnight on a rocking platform at 4 °C using the following dilutions: either 1:1000 for the antibody anti-LC3B (Cell Signaling Technology #2775S, Danvers, MA, USA), 1:4000 for anti-p62/SQSTM1 (Abnova #H00008878-M01, Walnut, CA, USA), and 1:10,000 for anti-β-actin (Sigma-Aldrich, #A5316, St. Louis, MO, USA). Primary antibodies were detected with the appropriate horseradish peroxidase-conjugated anti-mouse (Kirkegaard and Perry Laboratories #214-1806, Gaithersburg, MD, USA, dilution 1:5000) or anti-rabbit (Kirkegaard and Perry Laboratories #214-1516, Gaithersburg, MD, USA, dilution 1:5000) secondary antibodies and the ECL system (Thermo Fisher Scientific, Waltham, MA, USA).

### 4.12. Statistical Analysis

Data were expressed as the mean ± the standard error of the mean (SEM) of at least 3 independent experiments. Statistical analysis was carried out with GraphPad Prism software (San Diego, CA, USA) using one-way ANOVA with Bonferroni’s post-hoc test. Statistical significance was defined as *p* < 0.05.

## 5. Conclusions

**Figure 6:**
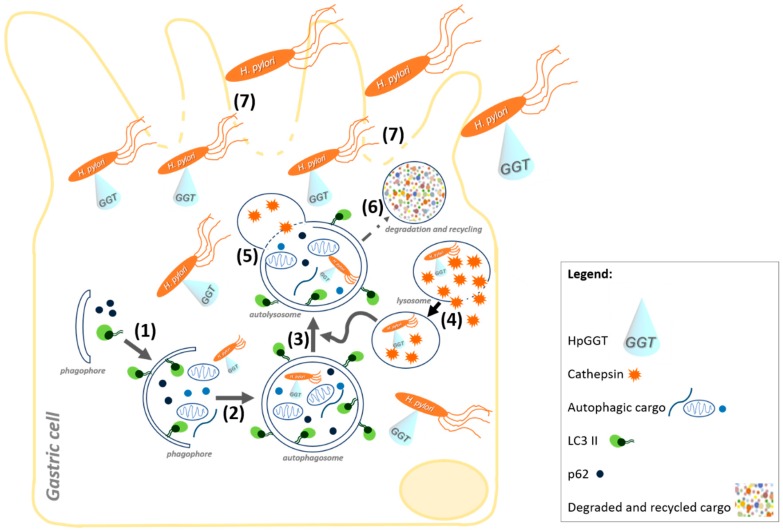
Schematic illustration depicting how the *Helicobacter pylori* (*H. pylori*) virulence factor gamma-glutamyltranspeptidase (HpGGT) acts upon infection of gastric (AGS and GES-1) cells. HpGGT is secreted and is shown to inhibit gastric cell autophagy (**1**–**6**) and, in the case of AGS cells, to do so by reducing levels of the lysosomal cysteine protease Cathepsin B. *H. pylori* is suggested in these cells to increase lysosomal membrane permeability to favor Cathepsin B leakage (**4**), thereby impairing lysosome degradation upon fusion with the autophagosome (**5**). In parallel, HpGGT is depicted as being necessary for *H. pylori* internalization (**7**) by gastric (AGS and GES-1) cells and thus as a virulence factor that facilitates bacterial invasion of gastric cells.

The present study points towards HpGGT as a novel autophagy inhibitor, which is also necessary to facilitate bacteria internalization in gastric cells. Our results are summarized in a working model (Figure 6), where HpGGT is depicted as a key virulence factor in the modulation of autophagy at late stages by decreasing lysosomal cathepsin B activity, in AGS cells. Furthermore, the new role for HpGGT in bacteria internalization suggests that this virulence factor could help the bacteria survive following treatments with antibiotics. Both effects together are expected to favor the progression of gastric cancer precursor lesions following *H. pylori* infection.

## Supplementary Materials

The following are available online at https://www.mdpi.com/2072-6694/11/6/801/s1. Figure S1: AGS infection with *H. pylori* wild type or the isogenic Hp∆ggt mutant induced to a similar extent the phosphorylation of AMPK and downstream targets of mTOR; Figure S2: Loss of HpGGT did not affect *H. pylori* adherence in gastric cells
