# Supplementary material for: A Novel Role for Helicobacter pylori Gamma-Glutamyltranspeptidase in Regulating Autophagy and Bacterial Internalization in Human Gastric Cells
Source: Cancers (Basel). 2019 Jun 10;11(6):801. doi: 10.3390/cancers11060801 (PMC6627848; doi:10.3390/cancers11060801)
Supplement: Supplementary file 1 [file cancers-11-00801-s001.pdf]

Supplementary Materials

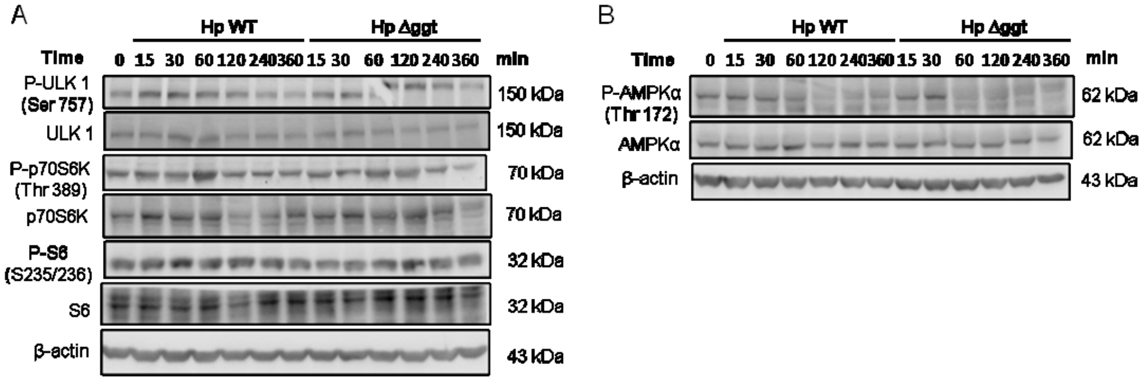

Figure S1. AGS infection with *H. pylori* wild type or the isogenic HpΔggt mutant induced to a similar extent the phosphorylation of AMPK and downstream targets of mTOR. Time course of ULK1 Ser 757 (**A**), p70S6K Thr 389 (**A**), S6 Ser 235/236 (**A**) and AMPK Thr 172 (**B**) phosphorylation in response to 6 h of infection with wild type *H. pylori* (HpWT) or the isogenic HpΔggt mutant was evaluated by western blotting. These blots are representative of three independent experiments.

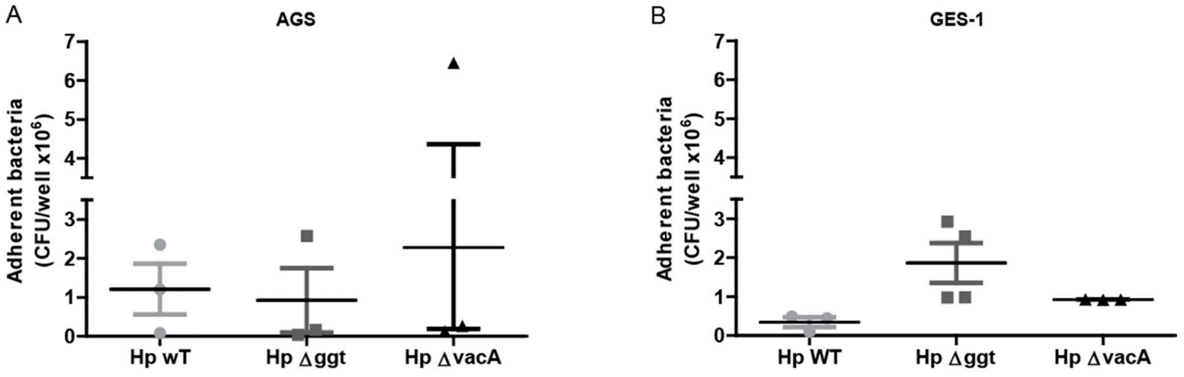

Figure S2. Loss of HpGGT did not affect *H. pylori* adherence in gastric cells. AGS and GES-1 cells were infected with *H. pylori* wild type (HpWT) and the isogenic mutants HpΔggt and HpΔvacA (MOI 100) for 6 h. After infection, non-adherent bacteria (cells treated with gentamicin 200 µg/mL for 1 h) and adherent bacteria were washed five times with PBS and lysed using saponin 0.1% for 15 min at 37 °C. Serial dilutions of the lysates were plated on blood agar, and adherent bacteria were counted as CFU in (**A**) AGS and (**B**) GES-1 cells. These data represent the mean (+SEM) of at least three independent experiments.
